# Pausing methotrexate improves immunogenicity of COVID-19 vaccination in elderly patients with rheumatic diseases

**DOI:** 10.1136/annrheumdis-2021-221876

**Published:** 2022-03-14

**Authors:** Amanthi Nadira Arumahandi de Silva, Leonie Maria Frommert, Fredrik N Albach, Jens Klotsche, Veronika Scholz, Lara Maria Jeworowski, Tatjana Schwarz, Alexander ten Hagen, Jan Zernicke, Victor Max Corman, Christian Drosten, Gerd-Rüdiger Burmester, Robert Biesen

**Affiliations:** 1 Department of Rheumatology and Clinical Immunology, Charité Universitätsmedizin Berlin, corporate member of Freie Universität Berlin and Humboldt-Universität, Berlin, Germany; 2 Epidemiology Unit, German Rheumatism Research Center Berlin – a Leibniz Institute (DRFZ), Berlin, Germany; 3 Institute of Virology, Charité Universitätsmedizin Berlin, Freie Universität Berlin and Humboldt-Universität zu Berlin, and German Centre for Infection Research (DZIF), Associated Partner Site, Berlin, Germany; 4 Labor Berlin, Charité - Vivantes GmbH, Berlin, Germany

**Keywords:** methotrexate, vaccination, autoimmune diseases, COVID-19

## Abstract

**Objective:**

To study the effect of methotrexate (MTX) and its discontinuation on the humoral immune response after COVID-19 vaccination in patients with autoimmune rheumatic diseases (AIRD).

**Methods:**

In this retrospective study, neutralising SARS-CoV-2 antibodies were measured after second vaccination in 64 patients with AIRD on MTX therapy, 31 of whom temporarily paused medication without a fixed regimen. The control group consisted of 21 patients with AIRD without immunosuppressive medication.

**Results:**

Patients on MTX showed a significantly lower mean antibody response compared with patients with AIRD without immunosuppressive therapy (71.8% vs 92.4%, p<0.001). For patients taking MTX, age correlated negatively with immune response (r=−0.49; p<0.001). All nine patients with antibody levels below the cut-off were older than 60 years. Patients who held MTX during at least one vaccination showed significantly higher mean neutralising antibody levels after second vaccination, compared with patients who continued MTX therapy during both vaccinations (83.1% vs 61.2%, p=0.001). This effect was particularly pronounced in patients older than 60 years (80.8% vs 51.9%, p=0.001). The impact of the time period after vaccination was greater than of the time before vaccination with the critical cut-off being 10 days.

**Conclusion:**

MTX reduces the immunogenicity of SARS-CoV-2 vaccination in an age-dependent manner. Our data further suggest that holding MTX for at least 10 days after vaccination significantly improves the antibody response in patients over 60 years of age.

**Key messages**

**What is already known about this subject?**

- Patients receiving methotrexate (MTX) have a reduced immune response after COVID-19 vaccination and holding MTX has shown to increase the immunogenicity after influenza vaccination.
- Yet, no previous studies have analysed the effect of MTX-hold for COVID-19 vaccination.

**What does this study add?**

- This study identified old age (≥60 years), short vaccine interval and MTX continuation as critical factors for an inadequate antibody response.
- We found a minimum of 10 days between vaccination and re-intake of MTX as the critical threshold to increase immunogenicity for patients ≥60 years of age.

**How might this impact on clinical practise or future developments?**

- Regarding ongoing booster vaccinations, our data suggest that especially older patients on MTX should hold MTX for at least 10 days after receiving a COVID-19 vaccination.

## Introduction

Until November 2021, SARS-CoV-2 had infected at least 250 million people worldwide and caused about 5 million deaths in a 23-month period.^1^ At the same time, enormous knowledge about SARS-CoV-2 and the related disease COVID-19 have been generated and the possibilities for prevention, diagnostics and treatments have improved remarkably.

Methotrexate (MTX) has been used for decades to treat a wide variety of immune-mediated diseases in oncology, rheumatology, dermatology, gastroenterology and neurology. Following prednisolone, MTX is the most prescribed anti-inflammatory drug worldwide with 1 million patients on MTX in the USA alone.^2^

Various immunosuppressants reduce the immune response after COVID-19 vaccination.^3^ Although several research groups have recently described a reduced vaccination response under MTX,^4 5^ in some cohorts MTX had no negative influence.^6 7^ Most of these studies did not collect data on whether or not patients had paused MTX during vaccinations, although more than one-third of patients had modified their medication on their own or on the advice of their rheumatologist, according to a recent survey.^8^ The discontinuation of immunosuppressive medication can improve the vaccination response as recently shown for mycophenolate.^9^

A reduced vaccination response under MTX was first described in 2016 for influenza vaccination.^10^ Follow-up data showed the increase in humoral immune response when pausing MTX 2 weeks before and after vaccination or only 2 weeks after vaccination.^11 12^ The time after and not before vaccination was decisive.^13^ However, data regarding MTX-hold during COVID-19 vaccination are still lacking, which is why current guidelines are based on experience with influenza vaccines, not considering mRNA-based technology used for COVID-19 vaccinations. Although current guidelines by the American College of Rheumatology as well as the German Society for Rheumatology recommend holding MTX 1–2 weeks after COVID-19 vaccination,^14 15^ the European League Against Rheumatism does not recommend pausing MTX.^16^

Therefore, our main objective was to study the effect of MTX and its discontinuation on the humoral immune response after COVID-19 vaccination in patients with autoimmune rheumatic diseases (AIRD). Secondary objective was to determine additional influencing factors on antibody response in these patients.

## Methods

### Study design and participants

This is a retrospective subanalysis of the VACCIMMUN study, which is an observational cohort study among patients with AIRD at the Charité Department for Rheumatology and Clinical Immunology in Berlin, Germany. Participants were recruited between April and September 2021 and had to meet the following inclusion criteria: age 18 years or older, AIRD diagnosis and vaccination with a COVID-19 vaccine authorised for use in Germany. For this analysis, only patients with AIRD under MTX therapy were considered, receiving either only MTX or MTX combined with low-dose prednisolone (defined as ≤5 mg/day), tumour necrosis factor-α inhibitors, hydroxychloroquine, leflunomide, interleukin (IL)-17 or IL-12/IL-23 inhibitors, since these immunosuppressive comedications are not known to have a remarkable impact on the immune response after vaccination.^15^ Additionally, patients with AIRD who were vaccinated under no immunosuppressive therapy served as controls. Information regarding medical history including COVID-19 vaccination status and immunosuppressive therapy were provided directly by patients and additionally validated with medical records. At the time of blood drawing, patients were asked about their MTX intake schedule around vaccinations. The decision on continuing or holding MTX was made by the patient or the attending physician and was only observed in the study. Patients who reported to have changed their MTX-intake schedule resulting in an MTX interval longer than 7 days around first or second vaccination were compared with patients who continued MTX therapy throughout both vaccinations.

### Laboratory analyses

Antibody response was measured predominantly about 2 weeks after the second dose of vaccination with maximum range from 11 to 112 days. Neutralising antibody levels were assessed using a surrogate virus neutralisation test (cPass Neutralisation, Medac, Wedel, Germany).^17^ Following the manufacturer’s protocol, patients who reached inhibition rates ≥30% were considered to have demonstrated a SARS-CoV-2-specific humoral response and are further defined as responders, while patients with inhibition rates <30% are defined as non-responders. Additionally, IgG antibodies against nucleocapsid, receptor binding domain (RBD), full spike and the S1 domain of the spike protein were tested using SeraSpot anti-SARS-CoV-2 IgG microarray-based immunoassay (Seramun Diagnostica, Heidesee, Germany) and served here for further validation purposes. Hence, all calculations were additionally performed using anti-RBD-IgG levels and can be found in the supplements. The threshold for reactivity for anti-SARS-CoV-2 IgG levels was set at >1.00 signal/cut-off in accordance with manufacturer’s protocol.

### Statistical analysis

Descriptive statistics included mean with SD and absolute and relative frequencies. The exact unconditional z-pooled test^18^ and χ² test were applied for binary and categorical data and the unpaired t-test with Welch’s correction for continuously distributed variables to perform hypotheses tests for group differences, as appropriate. The likelihood of response to vaccination was modelled by a Poisson generalised linear model with robust error variances and log link function including the covariates age, gender, MTX monotherapy, MTX in combination with prednisolone, MTX in combination with other disease-modifying antirheumatic drugs (DMARDs)±prednisolone, MTX-hold and vaccine interval as suggested by Zou.^19^ These covariates were selected based on the theoretical assumption that they could affect vaccination success and on the results of the univariate analysis. The association between antibody results (dependent variables anti-RBD-IgG concentrations or neutralising capacity) and the covariates age, gender, MTX monotherapy, MTX in combination with prednisolone and MTX in combination with other DMARDs±prednisolone, MTX-hold, vaccine interval and timing and duration of MTX-hold was estimated by a linear regression model. The unstandardised and standardised beta-coefficients were calculated for linear regression analyses in order to compare the strengths of association between parameters. The area under the curve (AUC) was calculated after fitting a logistic regression model to provide a measure of strengths of association for dichotomous outcomes. The Youden index was used to estimate thresholds for age and time of MTX break before and after vaccination from receiver operating characteristics (ROC). Statistical analyses were performed using GraphPad Prism V.9.2.0, R V.4.1.2 and STATA V.12.1.

### Patient and public involvement

This study aimed to provide evidence for future recommendations due to questions asked regarding MTX intake by patients and physicians. However, patients and the public were not directly involved in process of designing.

## Results

### Patient characteristics

Of 73 eligible patients receiving MTX, 9 were excluded due to unacceptable immunosuppressive comedication, irregular medication regimens and unclassifiable MTX-hold. The final cohort consisted of 64 patients with AIRD taking MTX (mean age 61 years, 70.3% women) and 21 patients with AIRD who did not receive any kind of immunosuppressive therapy as a control group (mean age 61, 90.5% women). Detailed clinical characterisation is given in online supplemental table 1. Patients in the no-therapy group were of similar age and body mass index (BMI), but more often female. They were less often diagnosed with rheumatoid arthritis and more often with systemic sclerosis.

10.1136/annrheumdis-2021-221876.supp1 Supplementary data

Of 64 patients on MTX, 31 patients reported to have held MTX for at least one vaccination (MTX-hold) while 33 patients had continued their MTX therapy without any interruption (MTX continued, table 1). Blood sampling occurred slightly earlier in the MTX-hold group than in the MTX continued group. There were no significant differences between these two groups regarding age, BMI, distribution of sex, vaccination regimes, diagnoses and immunosuppressive comedications (table 1).

**Table 1:** Characteristics of patients on MTX who held and continued MTX

	MTX continued (n=33)	MTX-hold (n=31)	MTX all (n=64)	P value*
Age, mean (SD)	62.4 (14.2)	59.6 (11.1)	61.1 (12.8)	0.391
Female, n (%)	21 (63.6)	24 (77.4)	45 (70.3)	0.251
BMI, mean (SD)	26.4 (4.52)	24.7 (3.30)	25.6 (4.03)	0.102
Rheumatic diagnosis				0.759
Rheumatoid arthritis, n (%)	21 (63.6)	23 (74.2)	44 (68.8)	
Psoriatic arthritis, n (%)	5 (15.2)	2 (6.5)	7 (10.9)	
Others, n (%)†	7 (21.2)	6 (19.4)	13 (20.3)	
Medication				0.553
MTX-mono, n (%)	14 (42.4)	12 (38.7)	26 (40.6)	
MTX+prednisolone, n (%)	7 (21.2)	5 (16.1)	12 (18.8)	
MTX+anti-TNF-α, n (%)‡	4 (12.1)	7 (22.6)	11 (17.2)	
MTX+anti-TNF-α+prednisolone, n (%)‡	5 (15.2)	2 (6.5)	7 (10.9)	
MTX+others, n (%)§	3 (9.1)	5 (16.1)	8 (12.5)	
Additional prednisolone, n (%)	12 (36.4)	8 (25.8)	20 (31.3)	0.377
Prednisolone dose (mg/day), mean (SD)	3.0 (1.8)	2.6 (1.1)	2.9 (1.6)	0.572
MTX dose (mg/week), mean (SD)	13.2 (4.5)	13.1 (4.1)	13.2 (4.3)	0.973
MTX oral application, n (%)	16 (48.5)	10 (32.3)	26 (40.6)	0.205
Vaccination				0.896
BNT162b2, n (%)	24 (72.7)	23 (74.2)	47 (73.4)	
mRNA-1273, n (%)	5 (15.2)	3 (9.7)	8 (12.5)	
AZD1222, n (%)	3 (9.1)	4 (12.9)	7 (10.9)	
AZD1222+BNT162b2, n (%)	1 (3.0)	1 (3.2)	2 (3.1)	
Vaccine interval in days, mean (SD)	39.0 (14.8)	41.9 (15.3)	40.4 (15.0)	0.444
Immune response				
Days from second vaccination, mean (SD)	35 (23)	28 (22)	32 (22)	0.237
Anti-RBD-IgG (S/CO), mean (SD)	3.7 (3.4)	6.3 (2.6)	5.0 (3.3)	**0.001**
Neutralising capacity (%), mean (SD)	61.2 (30.2)	83.1 (21.2)	71.8 (28.3)	**0.001**
Responders, neutralisation capacity, n (%)¶	25 (75.8)	30 (96.8)	55 (85.9)	**0.017**
Responders, anti-RBD-IgG response, n (%)**	21 (63.6)	30 (96.8)	51 (79.7)	**0.002**
MTX-hold				
For both vaccinations, n (%)	NA	24 (77.4)		
For only the first vaccination, n (%)	NA	2 (6.5)		
For only the second vaccination, n (%)	NA	5 (16.1)		
Duration of MTX-hold for first vaccination (days), mean (SD)	NA	15.1 (6.6)		
Duration of MTX-hold for second vaccination (days), mean (SD)	NA	16.9 (6.6)		

Significant results are in bold.

*P values compare MTX continued and MTX-hold and were calculated using the exact unconditional z-pooled test for binary variables (female, additional prednisolone, MTX oral application, responders neutralisation capacity, responders anti-RBD-IgG response), χ² test for categorical variables (rheumatic diagnosis, medication, vaccination) and unpaired t-test with Welch’s correction for continuous variables.

†For MTX continued: ANCA-associated vasculitis (n=1), axial spondyloarthritis (n=1), polymyalgia rheumatica (n=2), systemic sclerosis (n=1), myositis (n=1), systemic lupus erythematosus (n=1). For MTX-hold: axial spondyloarthritis (n=1), polymyalgia rheumatica (n=1), primary Sjögren’s syndrome (n=1), systemic sclerosis (n=2), myositis (n=1).

‡Adalimumab, certolizumab, etanercept, golimumab, infliximab.

§For MTX continued: hydroxychloroquine (n=1), secukinumab (IL-17 inhibitor, n=1), ustekinumab (IL-12/IL-23 inhibitor, n=1). For MTX-hold: hydroxychloroquine (n=1), leflunomide (n=2), leflunomide+prednisolone (n=1), secukinumab (IL-17 inhibitor, n=1).

¶Defined as neutralising capacity against SARS-CoV-2 ≥30%.

**Defined as anti-RBD-IgG levels >1.0 S/CO.

ANCA, antineutrophil cytoplasmic antibody; BMI, body mass index; IL, interleukin; MTX, methotrexate; NA, not available; S/CO, signal/cut-off; TNF, tumour necrosis factor.

### MTX reduces vaccination response

**Figure 1:**
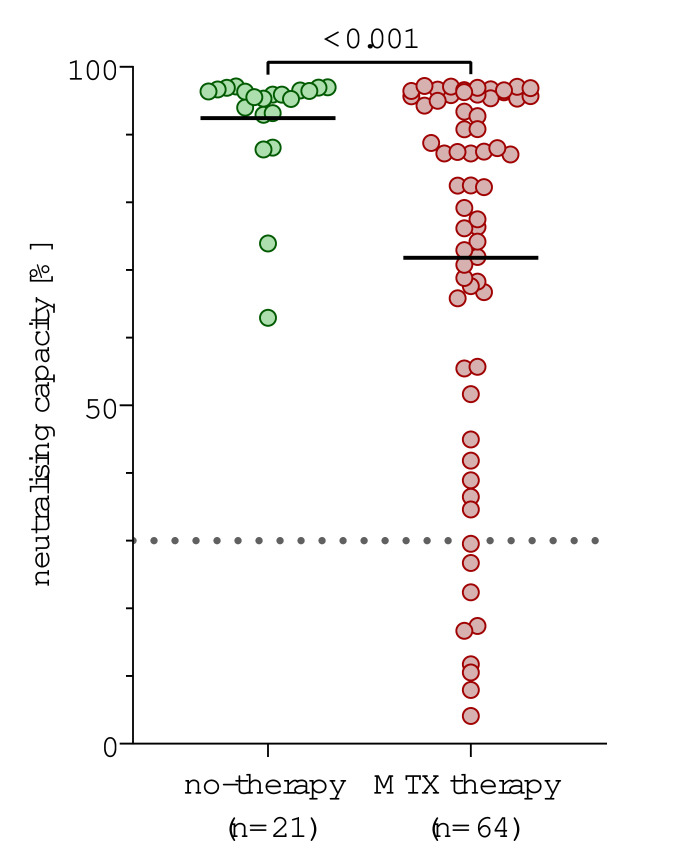
Comparison of neutralising capacity in patients with autoimmune rheumatic diseases (AIRD) without immunosuppression and with methotrexate (MTX) therapy. Neutralising capacity measured using surrogate virus neutralisation test after second vaccination in patients on MTX (n=64) represented by red dots versus patients with AIRD who were under no immunosuppressive therapy during both vaccinations (n=21) represented by green dots. P values were calculated using the parametric unpaired t-test with Welch’s correction.

Patients with AIRD without immunosuppressive therapy showed a significantly higher neutralising capacity (mean 92.4%, SD: 8.6) than patients with AIRD taking MTX (mean 71.8%, SD: 28.3, p<0.001, figure 1, online supplemental figure 1 for anti-RBD-IgG). This was still the case after adjusting for the possible confounders gender, age, vaccine regime and vaccine interval, AIRD diagnosis and duration from second vaccination to blood draw in a logistic regression (for neutralising capacity: beta=−19.5, 95% CI −31.4 to −7.7, p=0.002; for anti-RBD-IgG: beta=−1.61, 95% CI −3.03 to −0.18, p=0.028). None of the patients without immunosuppressive therapy were classified as non-responders (defined by neutralisation activity <30%), compared with 14.1% (n=9) among patients on MTX. Taking patients without immunosuppressive therapy in our cohort as a reference group for a typical antibody response after vaccination, the threshold for a not-altered inhibition rate could be set at 87.6% (AUC 0.75, Youden index 49.9). Accordingly, 38 of 64 patients on MTX (59.4%) demonstrated a lower antibody response after two vaccinations compared with an untreated group of patients with AIRD.

10.1136/annrheumdis-2021-221876.supp2 Supplementary data

### Factors influencing antibody response in patients on MTX

To identify factors influencing the antibody response under MTX, we compared COVID-19 vaccination responders (n=55, 85.9%) and non-responders (n=9, 14.1%) defined by neutralisation activity. Both groups were comparable in BMI, vaccine type, MTX application form, additional prednisolone intake, time of blood draw and immunosuppressive comedication (table 2). Dosage of MTX was not significantly associated with vaccination success (Spearman’s rank correlation, r=−0.02, p=0.867). However, a higher neutralisation capacity was significantly associated with young age, MTX-hold and female gender in univariate analysis (table 2) and multivariable analysis (table 3). If classification into responders and non-responders was based on anti-RBD-IgG results, 13 patients would fall into the non-responder group. While the effects of age and MTX-hold were still significant using anti-RBD-IgG levels, this was not the case for gender (online supplemental table 2, table 3). A longer vaccine interval was associated with an adequate humoral response to vaccination in our cohort (significant in t-test for neutralising capacity and anti-RBD-IgG levels; only significant in multivariable analysis for anti-RBD-IgG levels). In the following, we will analyse the effect of age and MTX-hold in more detail.

**Table 2:** Comparison of vaccination responders and non-responders among patients with AIRD taking MTX

	Responders* (n=55)	Non-responders (n=9)	P value†
Age, mean (SD)	59.5 (12.9)	70.3 (6.67)	**0.001**
Female, n (%)	42 (76.4)	3 (33.3)	**0.010**
BMI, mean (SD)	25.4 (4.09)	26.6 (3.70)	0.389
Medication			0.616
MTX-mono, n (%)	23 (41.8)	3 (33.3)	
MTX+prednisolone, n (%)	8 (14.5)	4 (44.4)	
MTX+anti-TNF-α, n (%)‡	10 (18.2)	1 (11.1)	
MTX+anti-TNF-α+prednisolone, n (%)‡	6 (10.9)	1 (11.1)	
MTX+HCQ, n (%)	2 (3.6)	0	
MTX+leflunomide, n (%)§	3 (5.5)	0	
MTX+anti-IL-17, n (%)¶	2 (3.6)	0	
MTX+anti-IL-12/IL-23, n (%)**	1 (1.8)	0	
MTX dose (mg/week), mean (SD)	13.0 (4.29)	14.2 (4.33)	0.469
MTX oral application, n (%)	25 (45.5)	1 (11.1)	0.057
Additional prednisolone, n (%)	15 (27.3)	5 (55.6)	0.103
Prednisolone dose (mg/day), mean (SD)	2.5 (1.4)	3.8 (1.6)	0.174
Vaccination			0.609
BNT162b2, n (%)	39 (70.9)	8 (88.9)	
mRNA-1273, n (%)	7 (12.7)	1 (11.1)	
AZD1222, n (%)	7 (12.7)	0	
AZD1222+BNT162b2, n (%)	2 (3.6)	0	
Vaccine interval in days, mean (SD)	42 (15)	31 (9)	**0.011**
Days from second vaccination, mean (SD)	30 (22)	40 (22)	0.259
MTX-hold, n (%)	30 (54.5)	1 (11.1)	**0.017**
For both vaccinations, n	23 (41.8)	1 (11.1)	
For only the first vaccination, n	2 (3.6)	0	
For only the second vaccination, n	5 (9.0)	0	

*Defined by neutralising capacity against SARS-CoV-2 ≥30%.

†P values were calculated using the exact unconditional z-pooled test for binary variables (female, MTX oral application, additional prednisolone, MTX-hold), χ² test for categorical variables (medication, vaccination) and unpaired t-test with Welch’s correction for continuous variables.

‡Adalimumab, certolizumab, etanercept, golimumab, infliximab.

§Additional low-dose prednisolone for n=1.

¶Secukinumab.

**Ustekinumab.

AIRD, autoimmune rheumatic diseases; BMI, body mass index; HCQ, hydroxychloroquine; IL, interleukin; MTX, methotrexate; TNF, tumour necrosis factor.

**Table 3:** Association of neutralising capacity and anti-RBD-IgG concentration with selected covariates in univariate and multivariable analyses (n=64)

	Univariate analysis				Multivariable analysis		
	RR*	P value	95% CI	AUC	RR*	P value	95% CI
Outcome: anti-RBD-IgG concentration >1 S/CO							
Female	1.18	0.280	0.88 to 1.58	0.60	1.23	0.125	0.94 to 1.62
Age (years)†	**0.93**	**<0.001**	**0.89 to 0.97**	**0.89**	**0.94**	**0.001**	**0.90 to 0.97**
MTX monotherapy	1.00			0.63	1.00		
MTX+prednisolone	0.79	0.284	0.51 to 1.22		0.86	0.415	0.60 to 1.24
MTX combination±prednisolone	1.05	0.687	0.84 to 1.30		1.04	0.693	0.86 to 1.25
MTX dose (mg)	0.99	0.688	0.97 to 1.02	0.54			
MTX-hold	**1.39**	**0.006**	**1.10 to 1.76**	**0.74**	**1.27**	**0.020**	**1.04 to 1.56**
Vaccine interval	**1.006**	**0.016**	**1.001 to 1.010**	**0.75**	**1.004**	**0.024**	**1.0006 to 1.008**
Outcome: neutralisation capacity ≥30%							
Female	1.36	0.055	0.99 to 1.87	0.72	**1.43**	**0.012**	**1.08 to 1.90**
Age (years)†	**0.96**	**0.008**	**0.92 to 0.99**	**0.77**	**0.96**	**0.018**	**0.93 to 0.99**
MTX monotherapy	1.00			0.68	1.00		
MTX+prednisolone	0.75	0.194	0.49 to 1.15		0.75	0.099	0.53 to 1.06
MTX combination±prednisolone	1.04	0.641	0.87 to 1.25		0.98	0.838	0.84 to 1.15
MTX dose (mg)	0.99	0.452	0.97 to 1.01	0.58			
MTX-hold	**1.28**	**0.019**	**1.04 to 1.57**	**0.72**	**1.17**	**0.039**	**1.00 to 1.38**
Vaccine interval	1.002	0.235	0.999 to 1.006	0.63	1.001	0.423	0.998 to 1.004

*RR was estimated by a Poisson generalised linear model with robust error variances and log link function in univariate and multivariable analyses according to Zou.^19^

†RR for increase by 5 years.

AUC, area under the curve; MTX, methotrexate; RR, relative risk; S/CO, signal/cut-off.

### Effect of MTX-hold and age

**Figure 2:**
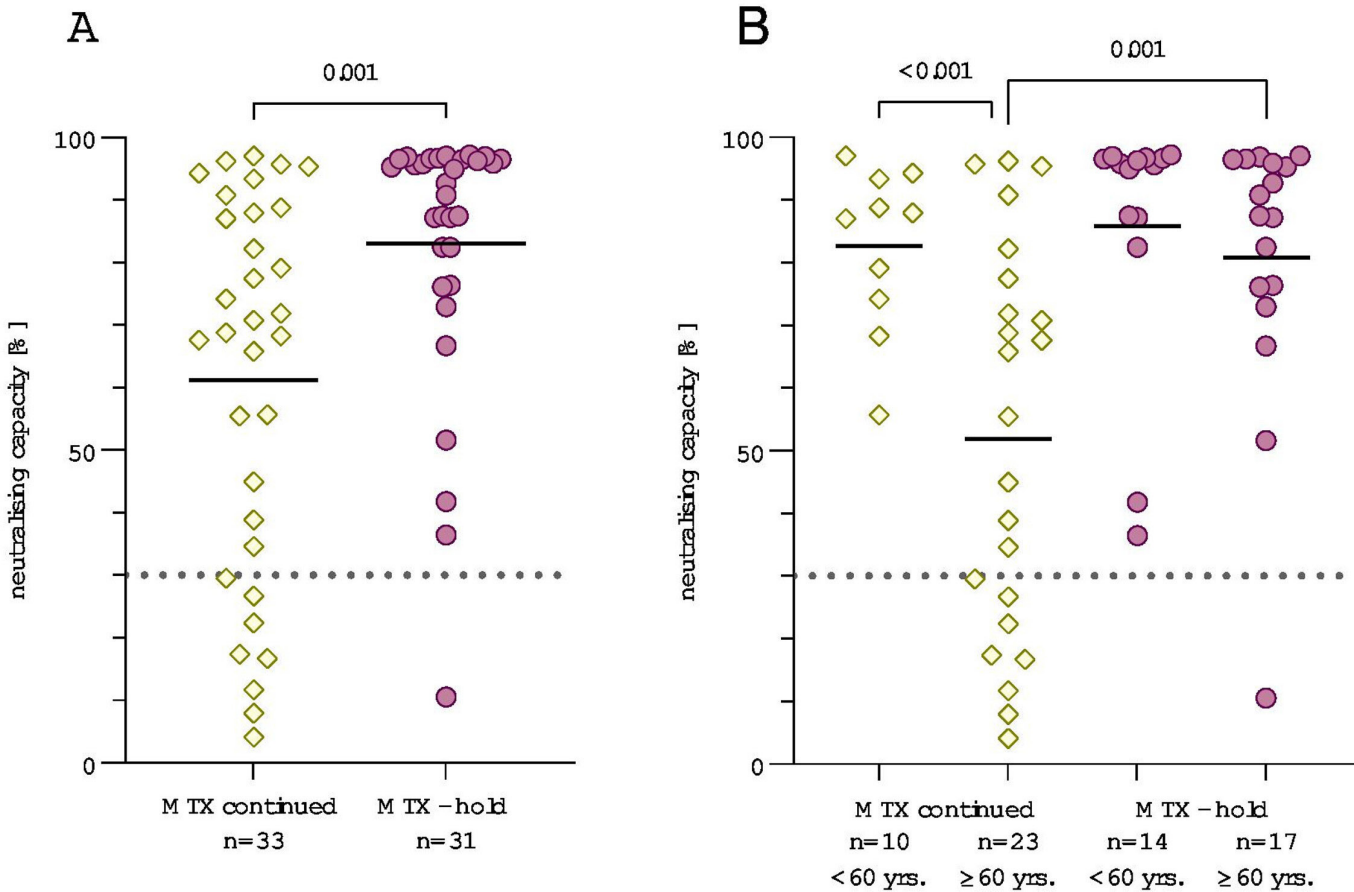
Comparison of patients with autoimmune rheumatic diseases (AIRD) who continued or held their methotrexate (MTX) during the COVID-19 vaccination. (A) Neutralising capacity measured using surrogate virus neutralisation test compared between patients who held MTX during vaccination (n=31) and patients who continued MTX therapy (n=33). (B) Neutralising capacity differentiated by age groups <60 years and ≥60 years. P values were calculated using the parametric unpaired t-test with Welch’s correction. Dotted line marks the cut-off value following manufacturer’s protocol (≥30%). Yellow squares represent patients who continued MTX therapy, purple dots represent patients who held MTX for at least one vaccination.

Patients who had changed their MTX intake schedule for at least one vaccination showed a significantly higher antibody response than patients who continued their MTX intake (p=0.001, figure 2A, online supplemental figure 2A for anti-RBD-IgG). Mean neutralisation was 61.2% for patients who continued their therapy and 83.1% for patients who held MTX (table 1). There was only one non-responder (3.2%) in the MTX-hold group, while there were eight non-responders (24.2%) in the MTX continued group. The effect of pause persisted in patients with MTX monotherapy, indicating that this effect cannot be explained by the existing comedication (online supplemental figure 3).

10.1136/annrheumdis-2021-221876.supp3 Supplementary data

10.1136/annrheumdis-2021-221876.supp4 Supplementary data

**Figure 3:**
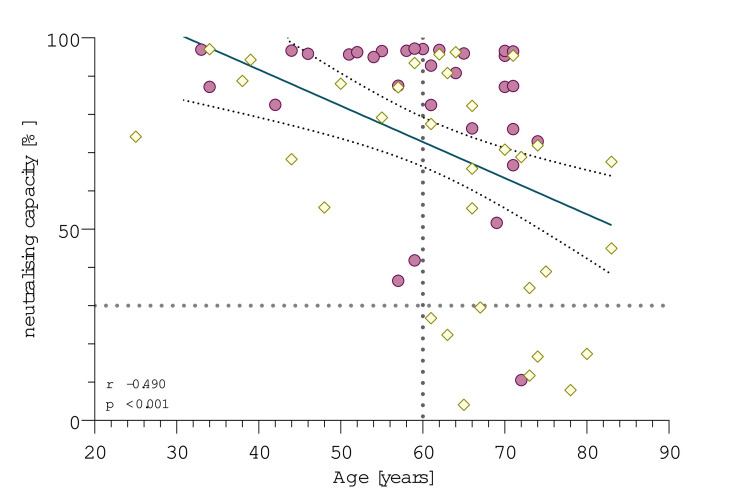
Correlation of age and neutralising capacity measured using surrogate virus neutralisation test. Purple dots represent patients who held methotrexate (MTX) during vaccination (n=31), yellow squares represent patients who continued MTX therapy (n=33). Neutralising antibodies were measured using a surrogate virus neutralisation test. Dotted lines mark the cut-off value following manufacturer’s protocol (≥30%) and the cut-off age used for further analysis at 60 years. P value and correlation coefficient were calculated using the Spearman’s rank correlation.

Vaccination response correlated significantly with age (Spearman’s rank correlation, −0.49, p<0.001, figure 3, online supplemental figure 4 for anti-RBD-IgG). No patient younger than 60 years was classified a non-responder which is why we further distinguished the MTX-hold and continued groups into patients older and younger than 60 years of age (figure 2B, online supplemental figure 2B for anti-RBD-IgG). Considering only patients who continued their MTX intake, patients ≥60 years of age (mean 51.9%) had a 30.7 percentage points lower mean inhibition rate than patients <60 years (mean 82.6%). Vice versa, neutralisation levels were 28.9 percentage points higher in patients older than 60 years who held MTX (mean 80.8%) compared with those who continued MTX (mean 51.9%). In contrast, when regarding patients under 60 years there were no significant differences in neutralisation rates between patients who held or continued MTX therapy.

10.1136/annrheumdis-2021-221876.supp5 Supplementary data

### Effect of timing and duration of MTX-hold

**Figure 4:**
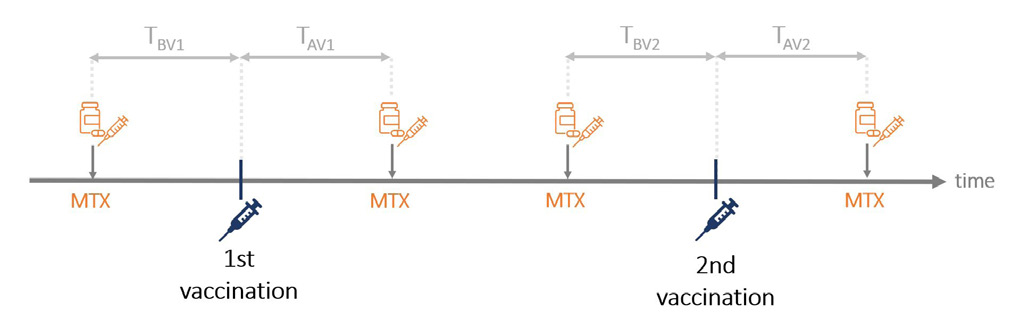
Visualisation of analysed time intervals. Time between methotrexate (MTX) intakes and COVID-19 vaccinations were assessed for each vaccination and added together to receive the total time before vaccinations (T_BV_=T_BV1_+T_BV2_) and after vaccinations (T_AV_=T_AV1_+T_AV2_). The MTX interval was defined as the total durations between two MTX intakes at the time of vaccination (T_AV_+T_BV_).

In the following, we considered all 64 patients and analysed the MTX interval at the time of vaccination, which was defined by the time between last MTX intake and vaccination (time before vaccination=T_BV_) and the time between vaccination and re-intake of MTX (time after vaccination=T_AV_, figure 4). One patient could not recall on which day MTX was taken and was therefore not considered for calculations of T_BV_ and T_AV_. We found that the duration of the MTX interval (T_BV_+T_AV_) significantly correlates with neutralising capacity (Spearman’s rank correlation, r=0.47, p<0.001). We further analysed which of these time periods is most likely to determine antibody response. By using linear regression analysis, we found time after vaccination (T_AV_) to be highly significant for adequate neutralisation rate and anti-RBD-IgG concentration in the elderly, but not for younger patients (table 4). Here, 10 days between vaccination and MTX re-intake (T_AV_) were determined as the critical cut-off based on the Youden index from ROC curve.

**Table 4:** Association of neutralising capacity and anti-RBD-IgG concentration with MTX intake timing using linear regression analysis (n=64)*

	All patients†				Patients <60 years‡				Patients ≥60 years‡			
	β	P value	95% CI	β_st_	β	P value	95% CI	β_st_	β	P value	95% CI	β_st_
Neutralisation capacity												
T_BV_	0.00	0.976	−0.20 to 0.19	0.00	−0.05	0.749	−0.37 to 0.27	−0.06	0.04	0.807	−0.26 to 0.33	0.03
T_AV_	**0.19**	**0.005**	**0.06 to 0.32**	**0.30**	0.09	0.301	−0.09 to 0.28	0.20	**0.24**	**0.012**	**0.06 to 0.43**	**0.32**
T_BV_ ≥10 days	−0.85	0.367	−2.73 to 1.02	−0.08	−0.36	0.789	−3.12 to 2.40	−0.05	−1.98	0.094	−4.31 to 0.35	−0.13
T_AV_ ≥10 days	**2.00**	**0.008**	**0.53 to 3.47**	**0.27**	−0.22	0.833	−2.34 to 1.91	−0.04	**2.91**	**0.001**	**1.29 to 4.53**	**0.36**
T_BV_+T_AV_	**0.13**	**0.035**	**0.01 to 0.25**	**0.25**	0.04	0.626	−0.13 to 0.22	0.11	**0.18**	**0.039**	**0.01 to 0.35**	**0.29**
Anti-RBD-IgG												
T_BV_	0.80	0.244	−0.56 to 2.15	0.09	0.47	0.575	−1.24 to 2.17	0.10	1.26	0.236	−0.86 to 3.39	0.12
T_AV_	**1.02**	**0.016**	**0.19 to 1.84**	**0.19**	0.11	0.739	−0.56 to 0.78	0.04	**1.63**	**0.036**	**0.12 to 3.14**	**0.22**
T_BV_ ≥10 days	−1.86	0.777	−14.96 to 11.24	−0.02	0.49	0.950	−15.49 to 16.47	0.01	−6.43	0.323	−19.43 to 6.57	−0.05
T_AV_ ≥10 days	**13.70**	**0.004**	**4.60 to 22.79**	**0.21**	−2.53	0.583	−11.96 to 6.90	−0.07	**20.03**	**0.005**	**6.57 to 33.50**	**0.26**
T_BV_+T_AV_	**0.95**	**0.003**	**0.33 to 1.58**	**0.22**	0.21	0.487	−0.41 to 0.84	0.09	**1.51**	**0.019**	**0.26 to 2.77**	**0.26**

Significant results are in bold.

*One patient who did not hold MTX could not recall on which exact day MTX was taken and was therefore only considered for calculations of T_BV_+T_AV_ (=7 days).

†Adjusted for female, age, MTX monotherapy, MTX+prednisolone, MTX combination±prednisolone, vaccine interval.

‡Adjusted for female, MTX monotherapy, MTX+prednisolone, MTX combination±prednisolone, vaccine interval. β (unstandardised beta-coefficient); β_st_ (standardised beta-coefficient); T_BV_ (time before vaccination), time between last MTX intake and vaccination; T_AV_ (time after vaccination), time between vaccination and re-intake of MTX; T_BV_+T_AV_, MTX interval at the time of vaccination.

MTX, methotrexate.

## Discussion

Our study found a reduced COVID-19 vaccination response in patients on MTX, demonstrates the effect of age and provides first data on the effect of MTX-hold around COVID-19 vaccinations.

Using neutralising capacity and the manufacturer’s cut-off, we found a slightly higher rate of vaccination responders among patients taking MTX (85.9%) than previously reported (47%–72%).^4 5^ Using ROC analysis and an untreated control group, we determined an adapted cut-off value and found adequate immune response in only 40.6% of patients on MTX. Hence, we confirmed the observations from previous studies that the antibody response is reduced under MTX therapy.^4 5^ In contrast, others described no effect of MTX on vaccination response.^6 7^ These varying results may be due to a lower effect size of MTX on vaccination response compared with other immunosuppressive therapies such as rituximab or mycophenolate, different test systems and statistical analyses used and other influencing factors such as age and pausing of MTX therapy.

We determined young age, MTX-hold and longer vaccine interval as the main factors improving antibody response after vaccination. The negative influence of age on vaccination response was already known.^20 21^ However, the consideration of age was not yet differentiated in previous studies investigating immune response under MTX therapy. Therefore, our data allow the assumption that continuous MTX intake and old age are potentiating negative factors. The positive effect of a longer vaccine interval on humoral immune response is in line with previously published works.^22 23^ These results were statistically significant in t-test for both antibody testing systems, but in the generalised linear model only for anti-RBD-IgG levels. This discrepancy is likely due to the higher statistical power of the t-test.

Patients who held MTX for at least one vaccination had a significantly higher immune response than those who continued MTX, which has not yet been described for COVID-19 vaccination. Nevertheless, our findings are in line with studies by Park *et al* investigating the effect of MTX-hold on the immune response to influenza vaccination.^11^ More detailed analysis showed that time after vaccination is crucial, which was also described by Park *et al* who recommended an MTX discontinuation of 2 weeks after influenza vaccination.^12 13^ In our study, we found a minimum time of 10 days after vaccination to be critical for immune response in patients ≥60 years. Additionally, the positive effect of MTX-hold was only statistically significant for patients 60 years or older. An effect also in younger patients might be observed in a larger cohort.

A strength of our study was that we validated all our neutralisation test results with an additional test system measuring anti-RBD-IgG levels. The latter defined four more patients as non-responders compared with the neutralisation test. This small number of conflicting test results is to be expected when using different test systems. The uneven distribution of gender among patients who had conflicting test results caused our analyses to suggest a significant influence of gender on the neutralisation result. This may be due to a statistical artefact and the effect of gender should be interpreted with caution.

This study has limitations. Since data regarding the MTX intake schedule during vaccination were assessed retrospectively, recall bias cannot be excluded. Due to our small sample size, we had to limit factors in the multivariable logistic regression modelling, which may lead to bias and residual confounding. For instance, confounding due to duration from vaccination to blood sampling, disease activity or AIRD diagnosis cannot with certainty be excluded in our analyses. We did not assess disease activity and safety of pausing MTX in our cohort, but current data do not indicate a significantly higher flare occurrence or disease activity in association with MTX discontinuation of 2 weeks.^24^ Also, T-cell response was not part of our study design. However, according to current studies, it can be assumed that measuring humoral vaccination response is an adequate mean to determine vaccine immunogenicity^25^ and that higher antibody levels correlate with a better clinical outcome.^26 27^ To address these limitations, a randomised controlled clinical trial to generate evidence for optimal management of MTX in COVID-19 vaccinations should be performed.

In conclusion, we present real-world data of clinical relevance regarding ongoing booster vaccinations. We determined age and MTX-hold as the main factors influencing antibody response during SARS-CoV-2 vaccinations and both aspects should be considered when discussing MTX regimens. Our data suggest that, if possible, patients older than 60 years of age should hold MTX for at least 10 days after receiving a COVID-19 vaccination.

## Data Availability

All data relevant to the study are included in the article. Data are available on reasonable request.
