# Supplementary material for: Pausing methotrexate improves immunogenicity of COVID-19 vaccination in elderly patients with rheumatic diseases
Source: Ann Rheum Dis. 2022 Mar 14;81(6):881–8. doi: 10.1136/annrheumdis-2021-221876 (PMC9120396; doi:10.1136/annrheumdis-2021-221876)
Supplement: Supplementary data [file annrheumdis-2021-221876supp001.pdf]

Supplementary table 1: Characteristics of MTX-patients and control-group

	**MTX-therapy (n=64)**	**No therapy (n=21)**	**All (n=85)**	**P value[*]**
Age, mean (SD)	61 (13)	61 (14)	61 (13)	0.899
Female, n (%)	45 (70.3)	19 (90.5)	64 (75.3)	0.069
BMI, mean (SD)	25.6 (4.0)	23.8 (3.9)	25.1 (4.1)	0.088
Rheumatic diagnosis				**<0.001**
Rheumatoid arthritis, n (%)	44 (68.6)	3 (14.3)	47 (55.3)	
Psoriatic arthritis, n (%)	7 (10.9)	2 (9.5)	9 (10.6)	
Systemic sclerosis, n (%)	3 (4.7)	8 (38.1)	11 (12.9)	
Others, n (%)[†]	10 (15.6)	8 (38.1)	18 (21.2)	
Vaccination				0.542
BNT162b2, n (%)	47 (73.4)	16 (76.2)	63 (74.1)	
mRNA-1273, n (%)	8 (12.5)	2 (9.5)	10 (11.8)	
AZD1222, n (%)	7 (10.9)	1 (4.8)	8 (9.4)	
AZD1222 + mRNA, n (%)[‡]	2 (3.1)	2 (9.5)	4 (4.7)	
vaccine interval in days, mean (SD)	40.4 (15.0)	38.6 (21.4)	40.0 (16.7)	0.725
Immune response				
days from 2nd vaccination, mean (SD)	32 (22)	31 (20)	32 (22)	0.807
Anti-RBD-IgG [S/CO], mean (SD)	5.0 (3.3)	6.8 (2.0)	5.4 (3.1)	**0.004**
Neutralising capacity [%], mean (SD)	71.8 (28.3)	92.4 (8.6)	76.9 (26.4)	**<0.001**
Responders, n (%)[§]	55 (85.9)	21 (100)	76 (89.4)	0.076

[*] P values were calculated using the exact unconditional z-pooled test for binary variables (female, responders), Chi^2 test for categorical variables (rheumatic diagnosis, vaccination) and unpaired t test with Welch's correction for continuous variables.
[†] For MTX group: ANCA-associated vasculitis (n=1), Axial spondyloarthritis (n=2), Polymyalgia rheumatica (n=3), Primary Sjogren's syndrome (n=1), Myositis (n=2), Systemic lupus erythematosus (n=1).
For no therapy group: Axial spondyloarthritis (n=2), Polymyalgia rheumatica (n=1), Primary Sjogren's syndrome (n=2), Systemic lupus erythematosus (n=1), Familial Mediterranean fever (n=1), peripheral spondyloarthritis (n=1).
[‡] MTX group: AZD1222 + BNT162b2 (n=2). No-therapy-group: AZD1222 + BNT162b2 (n=1), AZD1222 + mRNA-1273 (n=1).
[§] Defined by neutralising capacity against SARS-CoV-2 ≥ 30 %.
MTX, methotrexate.

Supplementary table 2: Comparison of vaccination responders and non-responders (anti-RBD-IgG levels)

	**Responders*** **(n=51)**	**Non-Responders** **(n=13)**	**P value†**
Age, mean (SD)	58 (12)	73 (7)	**<0.001**
Female, n (%)	38 (74.5)	7 (53.8)	0.163
BMI, mean (SD)	25.3 (3.9)	26.5 (4.7)	0.395
Medication			0.524
MTX-Mono, n (%)	22 (43.1)	4 (30.8)	
MTX+prednisolone, n (%)	7 (13.7)	5 (38.5)	
MTX+Anti-TNFα, n (%)‡	9 (17.6)	2 (15.4)	
MTX+Anti-TNFα+prednisolone, n (%)‡	5 (9.8)	2 (15.4)	
MTX+HCQ, n (%)	2 (3.9)	0	
MTX+Leflunomide, n (%)§	3 (5.9)	0	
MTX+Anti-IL-17, n (%)**	2 (3.9)	0	
MTX+Anti-IL-12/23, n (%)††	1 (2.0)	0	
MTX dose [mg/week], mean (SD)	13.2 (4.3)	13.1 (4.2)	0.935
MTX oral application, n (%)	23 (45.1)	3 (23.1)	0.187
Additional prednisolone, n (%)	13 (25.5)	7 (53.8)	0.054
Prednisolone dose [mg/day], mean (SD)	2.8 (1.3)	2.9 (2.0)	0.890
Vaccination			0.336
BNT162b2, n (%)	35 (68.6)	12 (92.3)	
mRNA-1273, n (%)	7 (13.7)	1 (7.7)	
AZD1222, n (%)	7 (13.7)	0	
AZD1222 + BNT162b2, n (%)	2 (3.9)	0	
vaccine interval in days, mean (SD)	43 (15)	30 (9)	**<0.001**
days from second vaccination, mean (SD)	30 (22)	39 (21)	0.174
MTX-hold, n (%)	30 (58.8)	1 (7.7)	**0.002**
for both vaccinations, n	23	1	/
for only the 1st vaccination, n	2	0	/
for only the 2nd vaccination, n	5	0	/

* Defined by anti-SARS-CoV-2-RBD-IgG>1.00 S/CO.
† P values were calculated using the exact unconditional z-pooled test for binary variables (female, MTX oral application, additional prednisolone, MTX hold), Chi² test for categorical variables (medication, vaccination) and unpaired t test with Welch's correction for continuous variables.
‡ Adalimumab, Certolizumab, Etanercept, Golimumab, Infliximab.
§ Additional lowdose prednisolone for n=1.
** Secukinumab.
†† Ustekinumab.
MTX, methotrexate; HCQ, hydroxychloroquine.

Figures

Supplementary figure 1: Comparison of anti-RBD-IgG-antibody levels in AIRD patients without immunosuppression and with MTX therapy. *Anti-RBD-IgG-antibody levels after second vaccination in MTX-patients (n=64) represented by red dots vs. patients who were under no immunosuppressive therapy during both vaccinations (n=21) represented by green dots. P values were calculated using the parametric unpaired t test with Welch's correction.*

Supplementary figure 2: Comparison of AIRD patients who continued or held their MTX during the COVID-19 vaccination. *(A) Anti-RBD-IgG concentrations compared between patients who held MTX during vaccination (n=33) and patients who continued MTX-therapy (n=31). (B) Anti-RBD-IgG concentrations differentiated by age groups <60 years and ≥60 years. P values were calculated using the parametric unpaired t test with Welch's correction. Dotted line marks the cut-off value following manufacturer's protocol (>1 S/CO). Yellow squares represent patients who continued MTX-therapy, purple dots represent patients who held MTX for at least one vaccination.*

Supplementary figure 3: Comparison of antibody response in AIRD patients without immunosuppressive medication and MTX monotherapy patients who continued (n=14) or held (n=12) their MTX during COVID-19 vaccination. *(A) Neutralising capacity measured using surrogate virus neutralisation test. Dotted line marks the cut-off value following manufacturer's protocol (≥30 %). (B) Anti-RBD-IgG-antibody levels after second COVID-19 vaccination. Dotted line marks the cut-off antibody concentration for adequate humoral immune response following manufacturer's protocol (>1 S/CO).*
P values were calculated using the parametric unpaired t test with Welch's correction. Yellow squares represent patients who continued MTX-therapy, purple dots represent patients who held MTX for at least one vaccination.

Supplementary figure 4: Correlation of Age and Anti-RBD-IgG-Antibody concentrations. *Purple dots represent patients who held MTX during vaccination (n=31), yellow squares represent patients who continued MTX-therapy (n=33). Dotted lines mark the cut-off antibody concentration for adequate humoral immune response following manufacturer's protocol (>1 S/CO) and the cut-off age observed for this cohort at 60 years. P value and correlation coefficient were calculated using the Spearman Rank correlation.*
